# Genome Sequence of *Pseudomonas* sp. Strain AN3A02, Isolated from Rhizosphere of Deschampsia antarctica Desv., with Antagonism against Botrytis cinerea

**DOI:** 10.1128/MRA.00320-20

**Published:** 2020-05-21

**Authors:** Matías Poblete-Morales, Claudia Rabert, Andrés F. Olea, Héctor Carrasco, Raúl Calderón, Gino Corsini, Evelyn Silva-Moreno

**Affiliations:** a Facultad de Ciencias de la Salud, Instituto de Ciencias Biomédicas, Universidad Autónoma de Chile, Santiago, Chile; b Instituto de Ciencias Químicas Aplicadas, Facultad de Ingeniería, Universidad Autónoma de Chile, Santiago, Chile; c Centro de Investigación en Recursos Naturales y Sustentabilidad, Universidad Bernardo O’Higgins, Santiago, Chile; d Instituto de Investigaciones Agropecuarias, INIA-La Platina, Santiago, Chile; University of Arizona

## Abstract

Here, we announce the draft genome sequence of *Pseudomonas* sp. strain AN3A02, isolated from the rhizosphere of one of the only two species of vascular plants existing in the Antarctic continent, Deschampsia antarctica Desv. This isolate, which inhibited the mycelial growth of Botrytis cinerea in dual culture, has a genome sequence of 6,778,644 bp, with a G+C content of 60.4%. These draft genome sequence data provide insight into the genetics underpinning the antifungal activity of this strain.

## ANNOUNCEMENT

Members of the genus *Pseudomonas* are widely spread in various environments and are rod-shaped, aerobic, flagellum mobile bacteria (1, 2). The genus comprises nonpathogenic and pathogenic species of plants, animals, and humans. Several members of the genus have been associated with the rhizosphere of various plants and have been shown to promote plant growth and antifungal activity (2–4); also, species of this genus have been studied for their potential applications in both agricultural and biotechnological settings, along with other areas (5, 6).

**FIG 1:**
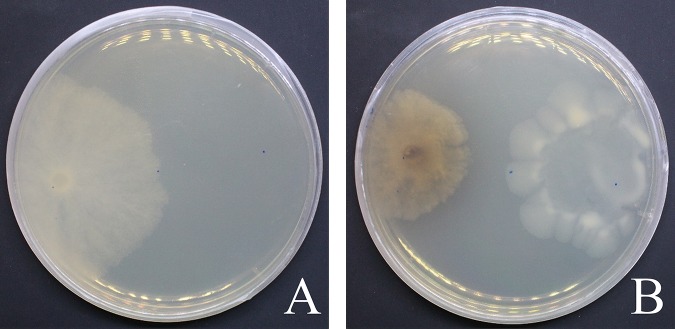
Antagonistic effects of *Pseudomonas* sp. strain AN3A02 against *B. cinerea* B05.10 in dual culture. (A) Control shows the growth of *B. cinerea* covering an NA plate; and (B) growth radius inhibition of *B. cinerea* in dual culture with AN3A02. Inoculation for 7 days at 22°C.

*Pseudomonas* sp. strain AN3A02 was isolated from rhizosphere samples of Deschampsia antarctica, obtained in the Antarctic summer of 2018 on King George Island (62°09′41″S, 58°28′10″W). For the isolation of cultivable bacteria, 200 mg of adhered root soil was inoculated in solid nutrient agar (NA) medium (BD) and incubated at 18°C for 72 hours. The bacterial isolation was conducted on a new plate with NA medium, and the pure culture stock was stored at −80°C in NA broth supplemented with 10 % (vol/vol) glycerol. According to the morphological characteristics and the biochemical profiles described (7), the isolate was classified as a *Pseudomonas* sp. that belongs to the fluorescent group. Screening was performed with the dual culture method, according to Rahman et al. (8) with modifications, in which an agar disk (6 mm) was taken from 4-day peptone-dextrose agar (PDA) culture plates of *Botrytis cinerea* B05.10 and placed on the periphery of an NA plate (90 mm). At the other end of the plate, a 10-μl drop of a suspension of *Pseudomonas* sp. AN3A02 (optical density [OD] at 620 nm of 0.1) was deposited on the opposite periphery of the same petri dish. As a control, *B. cinerea* B05.10 was similarly placed on the end NA plate. The cultures were incubated at 22°C for 7 days. A decrease in the diameter of the *B. cinerea* B05.10 colony was observed compared with that of the control, and the mean percentage of inhibition (±standard deviation) using the formula of Skidmore and Dickinson (9) was 54.3% ± 4.7% (Fig. 1).

For extraction of total genomic DNA, *Pseudomonas* sp. strain AN3A02 was cultured on solid agar plates of LB medium (BD) at 22°C for 24 hours from glycerol stock. One isolated colony was collected and resuspended in 1× phosphate-buffered saline (PBS), and the extraction was carried out with a NucleoSpin soil kit (Macherey and Nagel, Düren, Germany) according to the manufacturer’s protocols. The library synthesis was performed with the HyperPrep kit (Kapa Biosystems, Wilmington, MA, USA), and genomic sequencing with an Illumina MiSeq instrument was performed by Omega Bioservices (Norcross, GA, USA) with 2 × 300-bp paired-end (PE) reads. Default parameters were used for all software unless otherwise specified. A total of 8,721,132 reads were trimmed, normalized, and corrected with BBDuk and BBNorm Error version 38.37 prior to *de novo* assembly with Geneious Prime 2019.1 software. A total of 2,192,742 reads were used to assemble 20 scaffolds (*L*_50_, 3; *N*_50_, 585,680 bp) with an average depth of 63.8×. The length of the assembly is 6,778,644 bp, with a G+C content of 60.4%. Functional annotation performed using the Rapid Annotations using Subsystem Technology (RAST) server 2.0 (10) (http://rast.nmpdr.org/rast.cgi) via RASTtk (11) identified a total of 6,293 coding DNA sequences (CDS) and 66 RNAs. RAST annotation was used for most queries and annotations in the NCBI Prokaryotic Genome Annotation Pipeline (PGAP) (12), which is the public version available in NCBI.

This draft genome sequence report of *Pseudomonas* sp. AN3A02, isolated from rhizosphere samples of *D. antarctica*, confirms the presence of genes related to the acquisition of inorganic compounds and the alleviation of abiotic stress in plants and other essential characteristics of rhizobacteria. Using a series of *in silico* genome mining tools, a total of 11 secondary metabolite gene clusters were predicted by antiSMASH version 5 (13), including 5 nonribosomal peptide synthetase/polyketide synthase (NRPS/PKS) clusters; NapDos (14) predicted 7 KS domains and 22 C domains, while NP Searcher (15) identified 2 NRPS gene clusters.

It is important to note that this bacterium also possesses genes that encode molecules involved in the production of antimicrobial compounds, as well as the degradation and production of compounds that can participate in the protection and promotion of plant growth. Further in-depth analysis of this genome will increase our understanding of the antifungal activity of this strain for applications in biocontrol, will potentially lead to the discovery of new natural products, and will determine the potential *in vivo* effect of promoting the growth and protection of the plant.

### Data availability.

This whole-genome project for *Pseudomonas* sp. strain AN3A02 has been deposited in GenBank under the accession number JAARNI000000000. The BioProject number is PRJNA613530, the SRA number is SRR11362444, and the BioSample number is SAMN14408689.
